# Supplementary material for: Closer to critical resting-state neural dynamics in individuals with higher fluid intelligence
Source: Commun Biol. 2020 Feb 3;3:52. doi: 10.1038/s42003-020-0774-y (PMC6997374; doi:10.1038/s42003-020-0774-y)
Supplement: Supplementary file 1 — Supplementary Figures [file 42003_2020_774_MOESM1_ESM.pdf]

Supplementary Figures

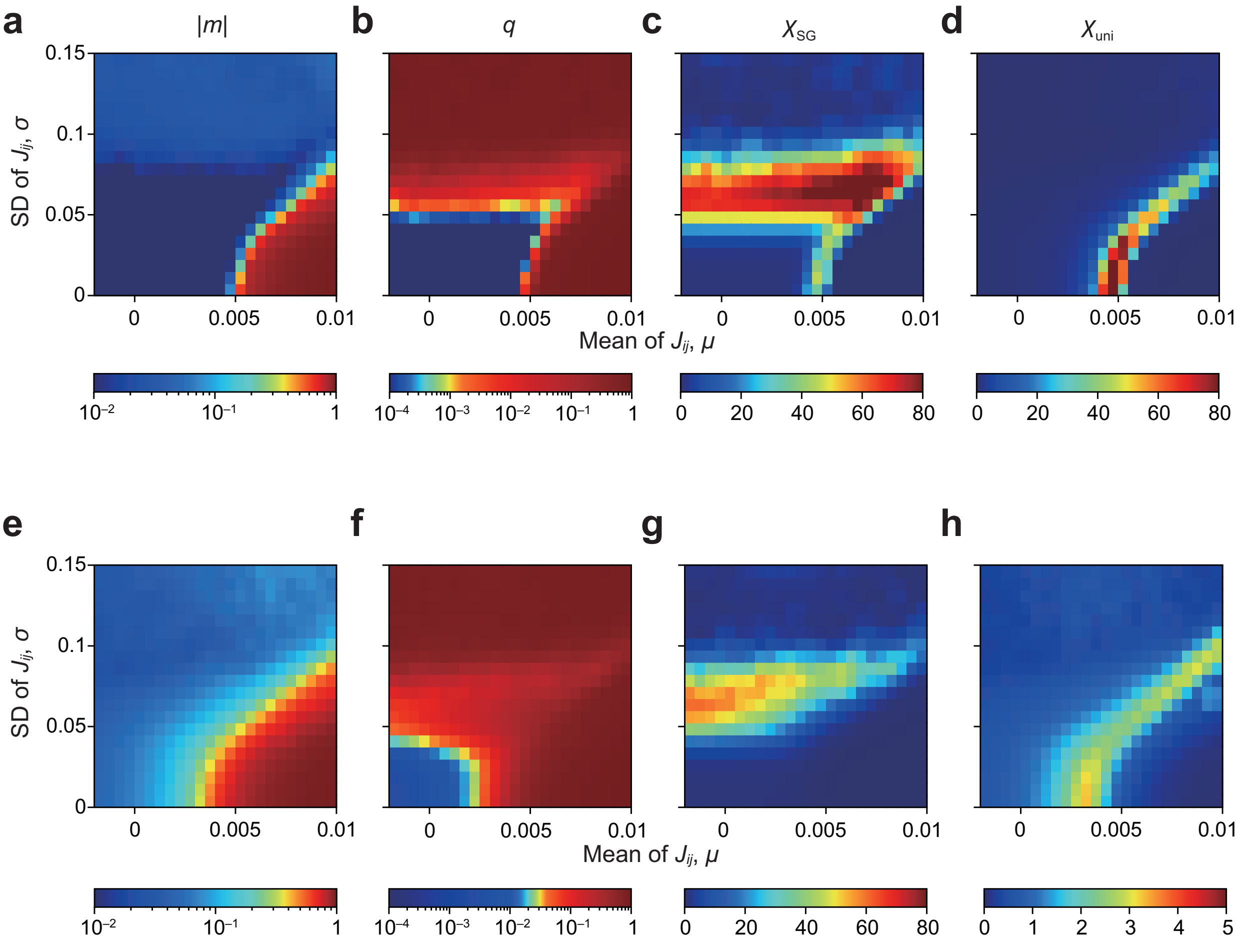

Supplementary Figure 1. Robustness of phase diagrams for the empirical data against variations in h. **(a–d)** $h_i = 0$ $(i = 1, \ldots, N)$. **(a–d)** $h_i = 2 \times \max_{1 \leq i' \leq N} \hat{h}_{i'}$ $(i = 1, \ldots, N)$. **a**, **e**: $|m|$. **b**, **f**: q. **c**, **g**: χ_{SG}. **d**, **h**: χ_{uni}. Note that the phase diagrams when $h_i = -2 \times \max_{1 \leq i' \leq N} \hat{h}_{i'}$ $(i = 1, \ldots, N)$ are the same as **(e–h)** owing to the symmetry. Therefore, given that **(a–d)** are qualitatively similar to **(e–h)**, we expect that h_i values satisfying $-2 \times \max_{1 \leq i' \leq N} \hat{h}_{i'} \leq h_i \leq 2 \times \max_{1 \leq i' \leq N} \hat{h}_{i'}$ produce qualitatively the same phase diagrams.

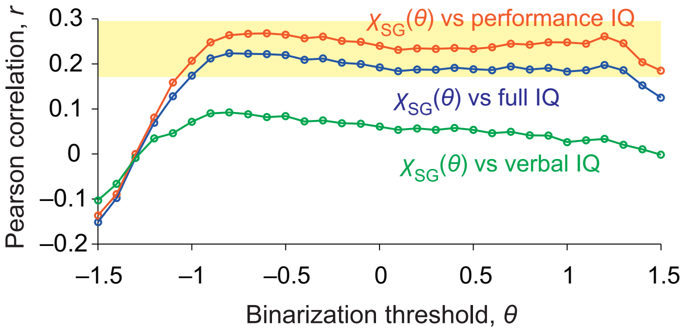

Supplementary Figure 2. Correlations between the IQ scores and $\chi_{\rm SG}(\theta)$ when the binarization threshold (i.e., θ) is varied between -1.5 and 1.5. We set $S_i(t) = +1$ if $z_i(t) \geq \theta$ and $S_i(t) = -1$ otherwise, extending Eq. (4) in the main text. As a guideline, with $\theta = -1$ and $\theta = 1$, the fraction of $S_i = +1$ is ≈ 0.853 and ≈ 0.148, respectively. The shaded area represents $P < 0.05$. Outliers were not removed in this analysis.

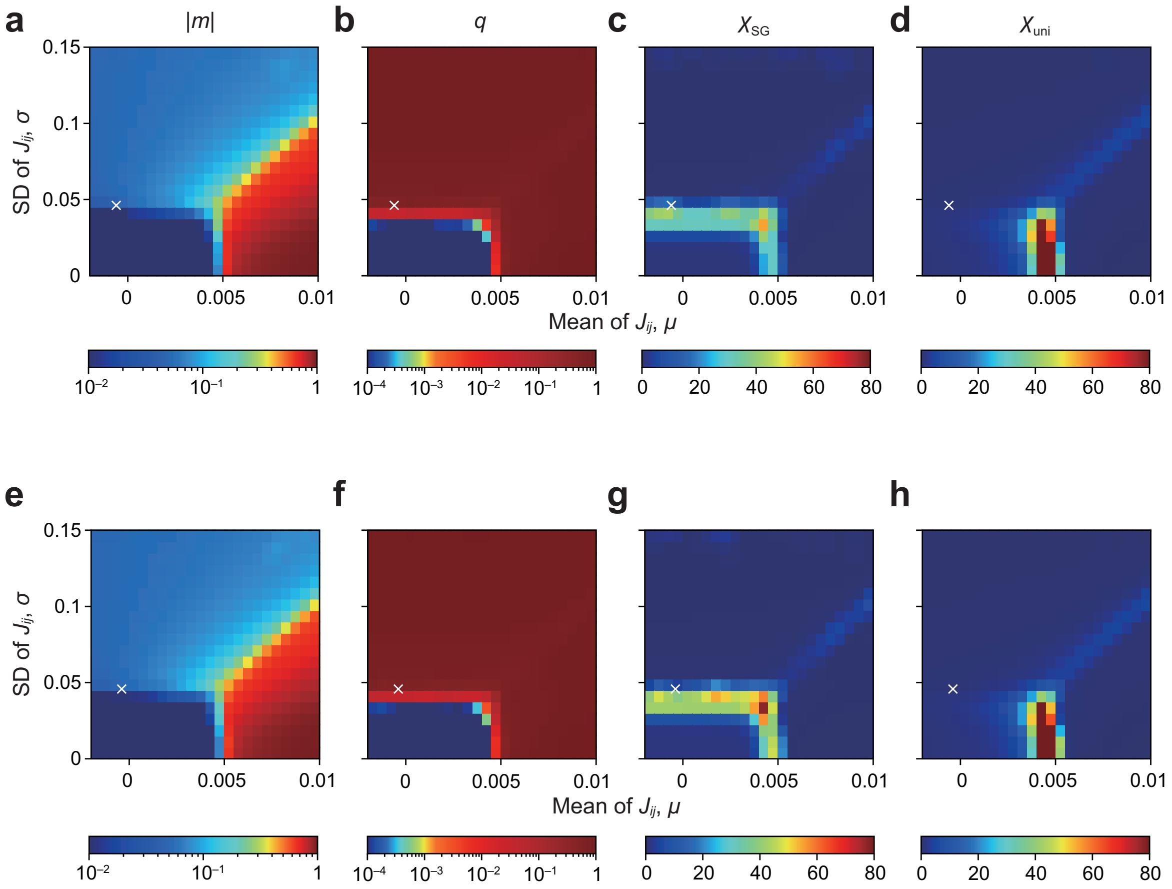

Supplementary Figure 3. Phase diagrams with different thresholds for binarization. We set $\theta = 1$ in (**a–d**) and $\theta = -1$ in (**e–h**). **a** and **e**: $|m|$. **b** and **f**: q. **c** and **g**: χ_{SG}. **d** and **h**: χ_{uni}.

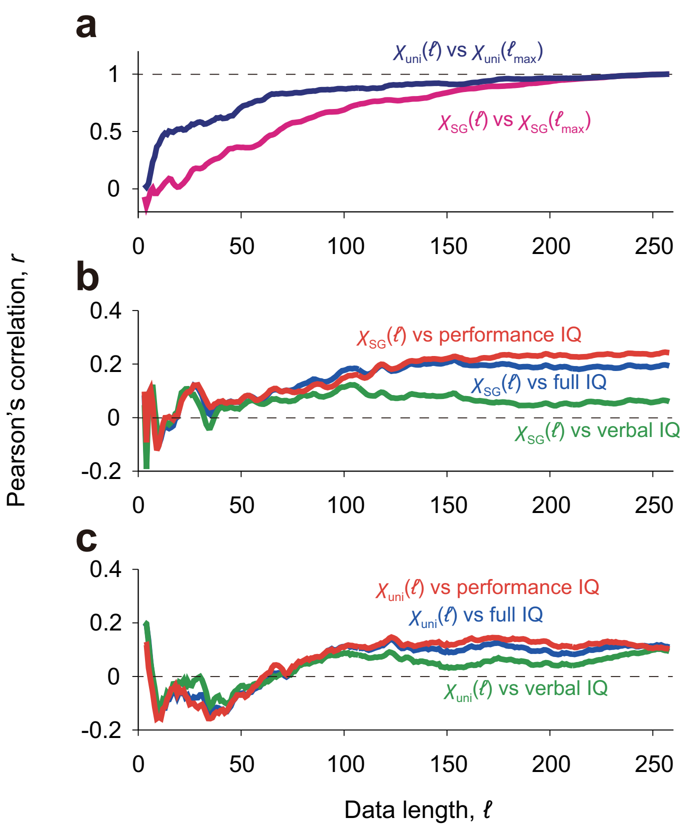

Supplementary Figure 4. Robustness of the spin-glass and uniform susceptibilities for the individual participants. We first computed χ_{SG} and χ_{uni} for each participant using the first ℓ volumes, and denote them by $\chi_{SG}(\ell)$ and $\chi_{uni}(\ell)$, respectively. For $\ell = \ell_{max} = 258$, these values (i.e., $\chi_{SG}(\ell_{max})$ and $\chi_{uni}(\ell_{max})$) coincide with the SG and uniform susceptibilities reported in the main text. **a** Pearson's correlation coefficient between $\chi_{SG}(\ell)$ and $\chi_{SG}(\ell_{max})$ and between $\chi_{uni}(\ell)$ and $\chi_{uni}(\ell_{max})$. **b** Pearson's correlation coefficient between $\chi_{SG}(\ell)$ and IQ scores. **c** Pearson's correlation coefficient between $\chi_{uni}(\ell)$ and IQ scores. To calculate the correlation coefficient, we regarded each participant as a sample.

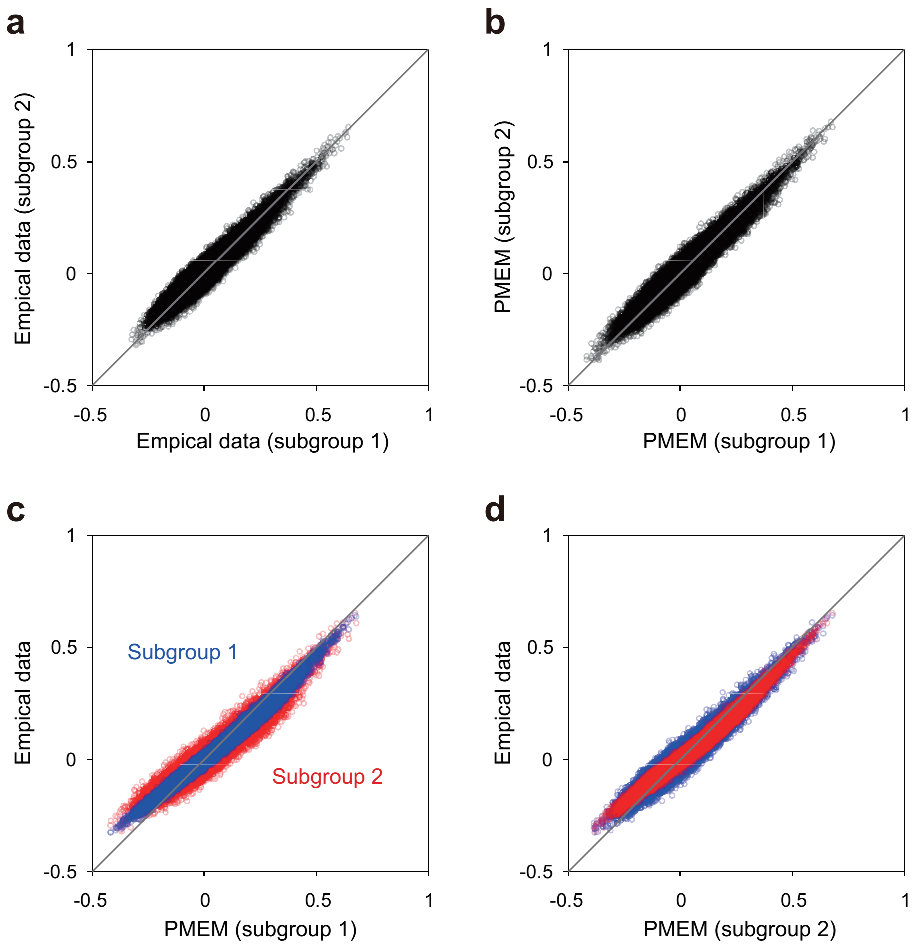

Supplementary Figure 5. Robustness of spin correlation, $\langle S_i S_j \rangle$, with respect to the grouping of participants. We split the participants into two halves, such that each subgroup of participants contains randomly selected 69 participants. **a** Comparison between $\langle S_i S_j \rangle$ directly calculated for the empirical data obtained from the first subgroup of participants and that for the second subgroup. A circle represents a pair of i and j $(1 \leq i < j \leq N)$. **b** Comparison between $\langle S_i S_j \rangle$ calculated for the PMEM that we estimated for the first subgroup and that for the second subgroup. **c** Comparison between $\langle S_i S_j \rangle$ calculated for the PMEM that we estimated for the first subgroup, shown on the horizontal axis, and $\langle S_i S_j \rangle$ directly calculated for the first or second subgroup of the empirical data, shown on the vertical axis. **d** Comparison between $\langle S_i S_j \rangle$ calculated for the PMEM that we estimated for the second subgroup and $\langle S_i S_j \rangle$ directly computed for the first or second subgroup of the empirical data. The solid lines represent the diagonal.

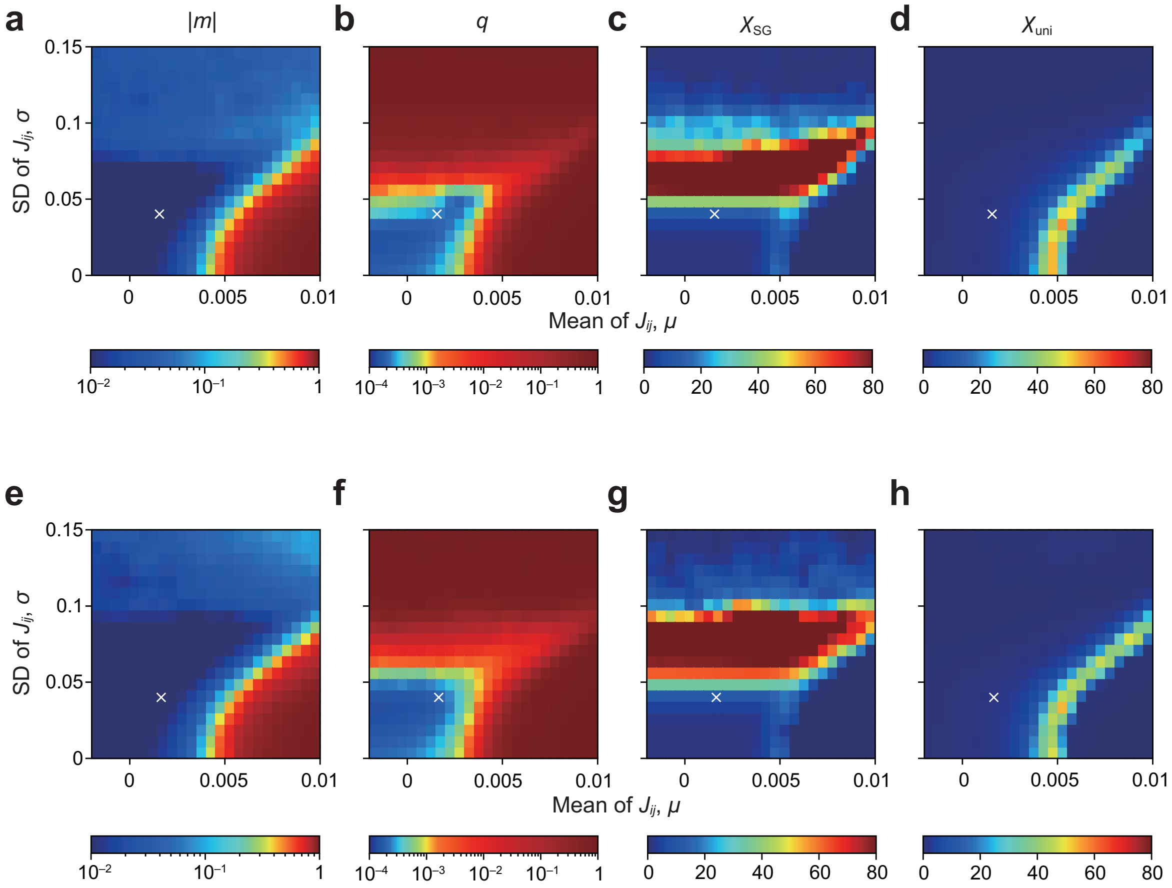

Supplementary Figure 6. Phase diagrams for each half of the participants. **a–d** Phase diagrams for the first subgroup of 69 participants used in Supplementary Fig. 5. **e–h** Phase diagrams for the second subgroup of 69 participants used in Supplementary Fig. 5. **a**, **e**: $|m|$. **b**, **f**: q. **c**, **g**: χ_{SG}. **d**, **h**: χ_{uni}. The crosses represent the mean and standard deviation of the J_{ij} estimated for the corresponding subgroup.

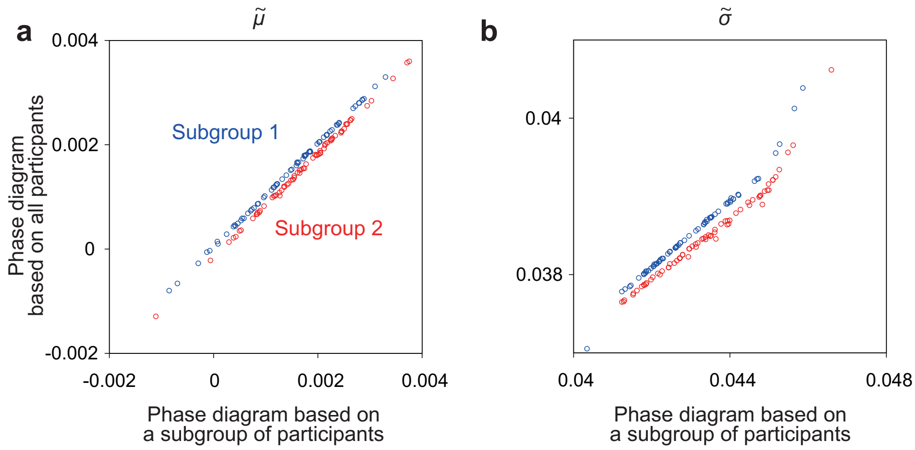

Supplementary Figure 7. Robustness of estimating $\tilde{\mu}$ and $\tilde{\sigma}$ for the individual participants. For each participant in each subgroup used in Supplementary Figs. 5 and 6, we computed $\tilde{\mu}$ (**a**) and $\tilde{\sigma}$ (**b**) using the phase diagrams of the corresponding subgroup composed of a half of the participants (i.e., 69 participants). Then, we compared the $\tilde{\mu}$ and $\tilde{\sigma}$ values thus calculated for the participant with those for the same participant computed using the phase diagrams based on all the participants (i.e., Fig. 1c,d in the main text). Each circle represents a participant. In (**b**), $\tilde{\sigma}$ estimated based on half the participants is consistently larger than that estimated based on all participants. This is because transition from the paramagnetic phase to the SG phase starts earlier (i.e., relevant order parameters start to increase) when all the participants are used as compared to when half the participants are used. This is considered to be a finite size effect in terms of the length of the data. However, we consider that this effect is not detrimental to our main results because, as shown in (**b**), the rank of the participants in terms of the $\tilde{\sigma}$ value is preserved with a high accuracy between the two cases.

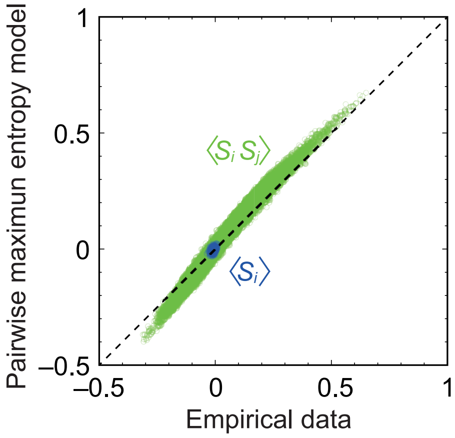

Supplementary Figure 8. Accuracy of the PL maximization. We compared $\langle S_i \rangle$ and $\langle S_i S_j \rangle$ between the empirical data and PMEM. Each circle represents an i value for $\langle S_i \rangle$ and an (i, j) pair for $\langle S_i S_j \rangle$. We set $h_i = 0$ ($i = 1, \ldots, N$) in the simulations. The results for PMEM were obtained by Monte Carlo simulations.

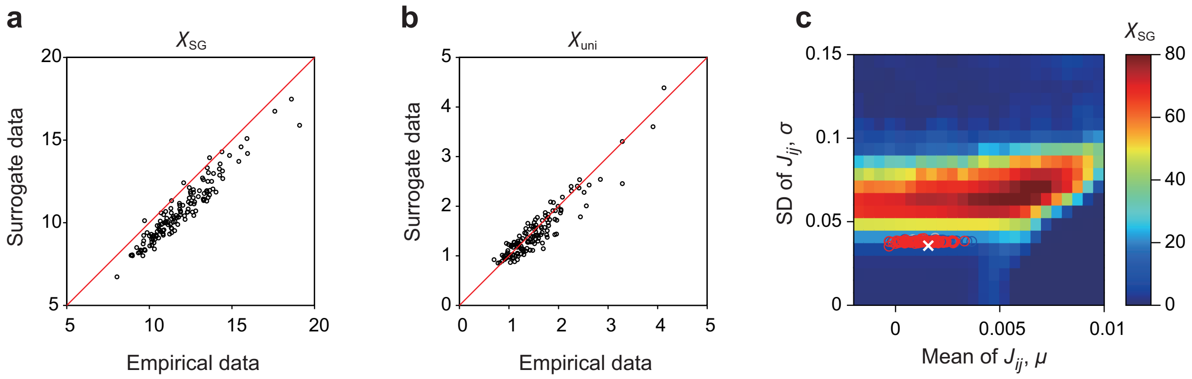

Supplementary Figure 9. Results obtained with surrogate data. **a** Comparison between χ_{SG} computed for the empirical data and that for the surrogate data. **b** Comparison between χ_{uni} computed for the empirical data and that for the surrogate data. **c** Distribution of the participants on the phase diagram computed for surrogate data (see also Fig. 2a in the main text). To obtain the surrogate data for each participant, we observed $t_{max} = 2 \times 10^4$ samples from a multivariate Gaussian distribution having the same mean vector and covariance matrix as those for the empirical data. Each circle represents a participant. These results suggest that our main results are reproduced solely from the covariance structure in the empirical data.

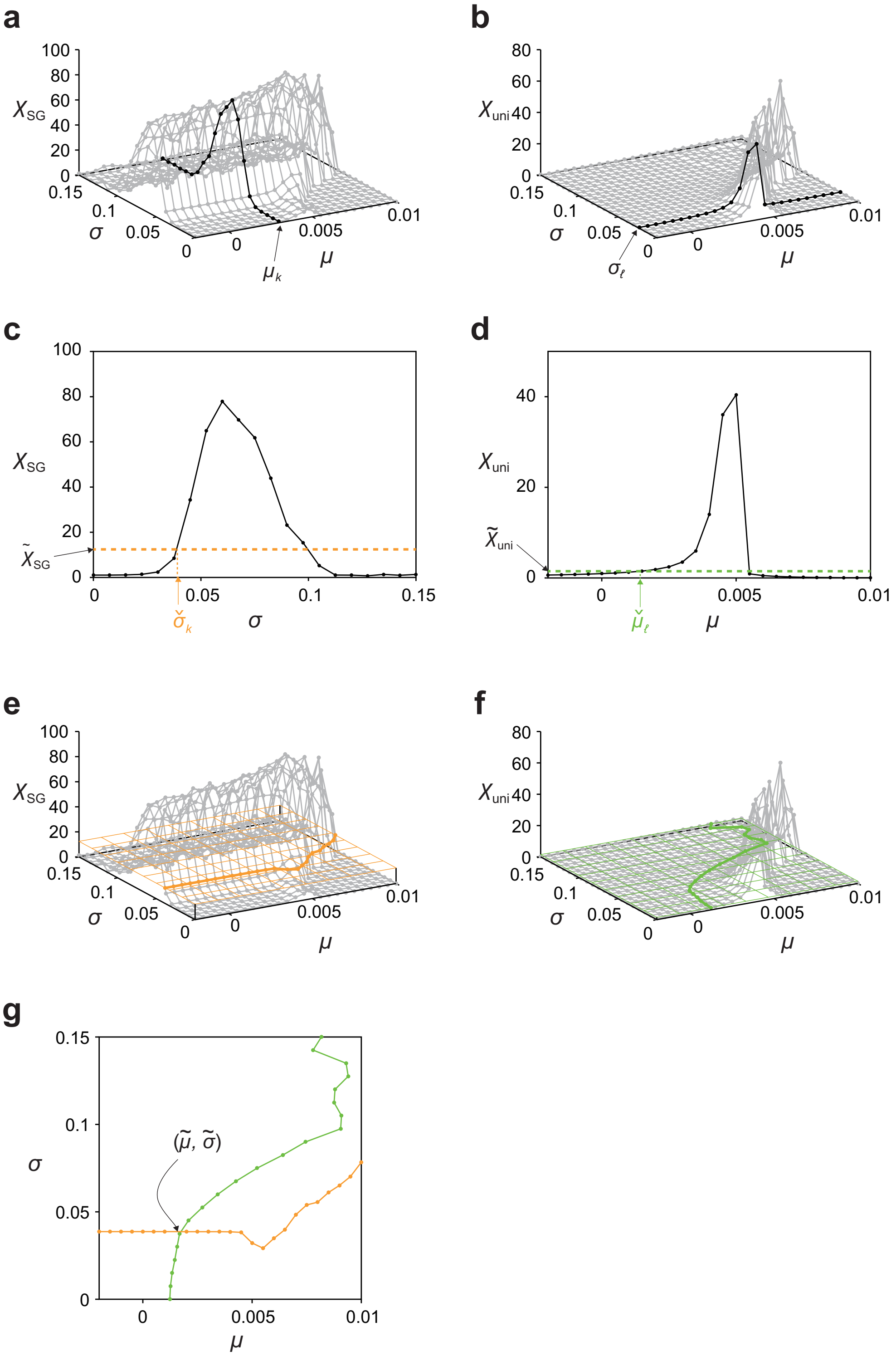

Supplementary Figure 10. Procedure for determining the μ and σ values for the individual participants. **a** Piecewise linear approximation of χ_{SG} as a function of σ for a given $\mu = \mu_k$. **b** Piecewise linear approximation of χ_{uni} as a function of μ for a given $\sigma = \sigma_\ell$. **c** By restricting oneself to the paramagnetic phase, one can identify the value of σ, denoted by $\breve{\sigma}_k$, that realizes $\chi_{\text{SG}}(\mu_k, \breve{\sigma}_k) \approx \tilde{\chi}_{\text{SG}}$. **d** By restricting oneself to the paramagnetic phase, one can identify the value of μ, denoted by $\breve{\mu}_\ell$, that realizes $\chi_{\text{uni}}(\breve{\mu}_\ell, \sigma_\ell) \approx \tilde{\chi}_{\text{uni}}$. **e** By connecting $(\mu_k, \breve{\sigma}_k)$ by a piecewise linear curve, we obtain a curve on which $\chi_{\text{SG}} \approx \tilde{\chi}_{\text{SG}}$. **f** By connecting $(\breve{\mu}_\ell, \sigma_\ell)$ by a piecewise linear curve, we obtain a curve on which $\chi_{\text{uni}} \approx \tilde{\chi}_{\text{uni}}$. **g** The intersection of the piecewise linear curves plotted in (**e**) and (**f**) yields the estimate of the μ and σ values for a single participant, denoted by $\tilde{\mu}$ and $\tilde{\sigma}$.

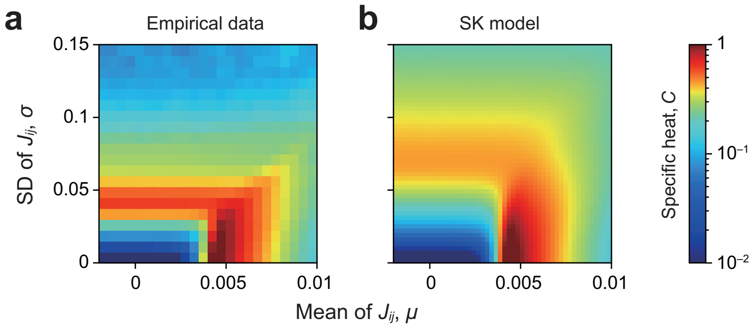

Supplementary Figure 11. Specific heat. **a** Empirical data, calculated for the entire population of the participants. **b** SK model.
