# Supplementary material for: Closer to critical resting-state neural dynamics in individuals with higher fluid intelligence
Source: Commun Biol. 2020 Feb 3;3:52. doi: 10.1038/s42003-020-0774-y (PMC6997374; doi:10.1038/s42003-020-0774-y)
Supplement: Supplementary file 3 — Reporting Summary [file 42003_2020_774_MOESM3_ESM.pdf]

nature research

Corresponding author(s): Naoki Masuda

Last updated by author(s): Jan 13, 2020

Reporting Summary

Nature Research wishes to improve the reproducibility of the work that we publish. This form provides structure for consistency and transparency in reporting. For further information on Nature Research policies, see Authors & Referees and the Editorial Policy Checklist.

Statistics

For all statistical analyses, confirm that the following items are present in the figure legend, table legend, main text, or Methods section.

n/a	Confirmed	
☐	☒	The exact sample size (n) for each experimental group/condition, given as a discrete number and unit of measurement
☐	☒	A statement on whether measurements were taken from distinct samples or whether the same sample was measured repeatedly
☐	☒	The statistical test(s) used AND whether they are one- or two-sided<br>*Only common tests should be described solely by name; describe more complex techniques in the Methods section.*
☐	☒	A description of all covariates tested
☐	☒	A description of any assumptions or corrections, such as tests of normality and adjustment for multiple comparisons
☐	☒	A full description of the statistical parameters including central tendency (e.g. means) or other basic estimates (e.g. regression coefficient) AND variation (e.g. standard deviation) or associated estimates of uncertainty (e.g. confidence intervals)
☐	☒	For null hypothesis testing, the test statistic (e.g. F, t, r) with confidence intervals, effect sizes, degrees of freedom and P value noted<br>*Give P values as exact values whenever suitable.*
☒	☐	For Bayesian analysis, information on the choice of priors and Markov chain Monte Carlo settings
☒	☐	For hierarchical and complex designs, identification of the appropriate level for tests and full reporting of outcomes
☐	☒	Estimates of effect sizes (e.g. Cohen's d, Pearson's r), indicating how they were calculated

Our web collection on statistics for biologists contains articles on many of the points above.

Software and code

Policy information about availability of computer code

Data collection	No software was used for data collection.
Data analysis	We used FMRIB's Software Library (FSL; www.fmrib.ox.ac.uk/fsl) for preprocessing.

For manuscripts utilizing custom algorithms or software that are central to the research but not yet described in published literature, software must be made available to editors/reviewers. We strongly encourage code deposition in a community repository (e.g. GitHub). See the Nature Research guidelines for submitting code & software for further information.

Data

Policy information about availability of data

All manuscripts must include a data availability statement. This statement should provide the following information, where applicable:

- Accession codes, unique identifiers, or web links for publicly available datasets
- A list of figures that have associated raw data
- A description of any restrictions on data availability

We used data from a publicly available data set (Nathan Kline Institute's (NKI) Rockland phase I Sample; http://fcon_1000.proj- ects.nitrc.org/indi/pro/nki.html)

Field-specific reporting

Please select the one below that is the best fit for your research. If you are not sure, read the appropriate sections before making your selection.

☒ Life sciences ☐ Behavioural & social sciences ☐ Ecological, evolutionary & environmental sciences

For a reference copy of the document with all sections, see nature.com/documents/nr-reporting-summary-flat.pdf

Life sciences study design

All studies must disclose on these points even when the disclosure is negative.

Sample size	No sample size calculations were performed.
Data exclusions	No data were excluded from the analyses.
Replication	We did not perform replication analyses using an independent data set. We confirmed that our results were replicated using the two halves of the data set.
Randomization	Our analyses were performed on a single group of participants. In Fig. 2, we split the participants into two groups based on the performance IQ score without randomization to examine the effect of the performance IQ. In Supplementary Figures 5, 6, and 7, participants were randomly split into two halves to confirm the reproducibility of our results.
Blinding	Blinding was not relevant as we analyzed a single group of participants.

Reporting for specific materials, systems and methods

We require information from authors about some types of materials, experimental systems and methods used in many studies. Here, indicate whether each material, system or method listed is relevant to your study. If you are not sure if a list item applies to your research, read the appropriate section before selecting a response.

Materials & experimental systems

n/a	Involved in the study	
☒	☐	Antibodies
☒	☐	Eukaryotic cell lines
☒	☐	Palaeontology
☒	☐	Animals and other organisms
☐	☒	Human research participants
☒	☐	Clinical data

Methods

n/a	Involved in the study	
☒	☐	ChIP-seq
☒	☐	Flow cytometry
☐	☒	MRI-based neuroimaging

Human research participants

Policy information about studies involving human research participants

Population characteristics	138 healthy adults (ages 18 - 85, 54 females and 84 males)
Recruitment	A convenience sample consisting of individuals who were willing to participate in the study was used. The representativeness of the sample may be diminished by the self-selected bias, which is not considered to be a significant confounding factor in our results.
Ethics oversight	The study was approved by the institutional review board of the Nathan Kline Institute. Written informed consent was obtained from all the participants.

Note that full information on the approval of the study protocol must also be provided in the manuscript.

Magnetic resonance imaging

Experimental design

Design type	Resting state
Design specifications	The duration of each fMRI run was 10 m 55 s (TR=2500 ms).
Behavioral performance measures	No behavioral measures were acquired during the fMRI scan recordings.

Acquisition

Imaging type(s)	Functional, Structural
Field strength	3T
Sequence & imaging parameters	fMRI data were obtained with an echo planner imaging (EPI) sequence (TE=30 ms, flip angle=80, FOV=216 ms, spatial

Sequence & imaging parameters	resolution = 3 mm isotropic). Anatomical images were acquired with T1-weighted sequence (MPRAGE; TR=2,500 ms, TE=3.5 ms, flip angle=8, spatial resolution = 1 mm isotropic).
Area of acquisition	Whole brain scans

Diffusion MRI ☐ Used ☒ Not used

Preprocessing

Preprocessing software	FMRIB's Software Library (FSL; www.fmrib.ox.ac.uk/fsl)
Normalization	Normalization was performed with FLIRT
Normalization template	We used the standard Montreal Neurological Institute (MNI) 2-mm brain.
Noise and artifact removal	We regressed out six head motion parameters, global signal, cerebrospinal fluid (CSF) signal, and white matter (WM) signal with FSL FEAT. We then applied band-pass temporal filtering (0.01–0.1 Hz).
Volume censoring	No volume was excluded using motion censoring.

Statistical modeling & inference

Model type and settings	No statistical modeling was used in the first and second level analyses.
Effect(s) tested	Does not apply.

Specify type of analysis: ☐ Whole brain ☒ ROI-based ☐ Both

Anatomical location(s): We used ROI coordinates defined by Power et al. 2011.

Statistic type for inference (See Eklund et al. 2016)	Does not apply.
Correction	Does not apply.

Models & analysis

n/a	Involved in the study
☒	☐ Functional and/or effective connectivity
☒	☐ Graph analysis
☒	☐ Multivariate modeling or predictive analysis
